# Morganella morganii Bacteremia in an Immunocompetent 13-Year-Old Male: A Rare Pediatric Case

**DOI:** 10.7759/cureus.91963

**Published:** 2025-09-10

**Authors:** Noah B Manz, Alicia A Briggs

**Affiliations:** 1 Pediatrics, Larner College of Medicine, University of Vermont, Burlington, USA; 2 Pediatrics, Connecticut Children’s Medical Center, Hartford, USA

**Keywords:** bacteremia, immunocompetent host, morganella morganii, multidrug-resistant organisms, urinary tract infection

## Abstract

*Morganella morganii* is a gram-negative bacillus with intrinsic resistance to many β-lactam antibiotics, including penicillins and first-/second-generation cephalosporins. It is frequently associated with urinary tract, wound, and respiratory infections, and can develop into bacteremia in older adults due to age-related comorbidities and immunosenescence, but this has rarely been described in children. In this report, we present the case of a 13-year-old male with a urinary tract infection complicated by *M. morganii* bacteremia, who was successfully treated with antibiotics. To our knowledge, this is one of the few documented cases of *M. morganii* bacteremia in an immunocompetent pediatric patient.

## Introduction

*Morganella morganii* is a facultatively anaerobic, gram-negative bacillus belonging to the Proteeae group within the Enterobacterales family [1]. It is commonly found in the human gastrointestinal tract and is increasingly recognized as a cause of both community- and hospital-acquired infections [2-4].

Like other Proteeae, *M. morganii* has intrinsic resistance to many β-lactam antibiotics due to chromosomally encoded AmpC β-lactamases, and some strains may also acquire extended-spectrum β-lactamases (ESBLs) [1]. This resistance profile makes the organism epidemiologically important, although aspects of its virulence and disease characteristics remain less well understood [3,4].

Clinical reports most frequently describe urinary tract infections (UTIs), with generally favorable outcomes [5]; however, progression to bacteremia and sepsis has been documented. A recent population-based surveillance study reported an annual incidence of *M. morganii* bacteremia of 9.2 cases per million, predominantly affecting adults (median age: 71 years), with an associated mortality of 21% [4].

Earlier estimates placed the incidence at approximately 3 per million annually, with >80% of affected patients having at least one comorbid condition, most commonly diabetes, renal disease, or congestive heart failure [6]. These studies have predominantly described adult populations, particularly older adults with comorbidities.

Pediatric cases remain exceedingly rare and, when reported, are usually in children with significant comorbid conditions or in neonates with immune system immaturity [7-11]. To our knowledge, this case represents one of the few documented instances of *M. morganii* bacteremia in an immunocompetent pediatric patient.

## Case presentation

A 13-year-old male was brought to the Emergency Department in April 2025 for sudden-onset lethargy, fever with chills, nausea, vomiting, and headache, without signs of meningismus. Upon arrival, he was febrile and appeared fatigued and pale, but oriented to time and place. Vital signs were: T 39.5°C, HR 140 bpm, BP 99/65 mmHg, RR 15, and SpO₂ 99% on room air. His past medical history was significant for myelomeningocele (repaired at birth), hydrocephalus with a ventriculoperitoneal shunt, paraplegia, neurogenic bladder managed with frequent straight catheterization, colostomy, and UTIs with multidrug-resistant organisms, including *Pseudomonas* and ESBL-producing *Escherichia coli*. There were no signs of shunt malfunction on physical examination, and neurological findings were consistent with his baseline lower extremity paralysis. A workup for sepsis was subsequently initiated, and the patient received a fluid bolus and empiric ceftriaxone for presumed UTI. Blood cultures, urinalysis, chest X-ray, and a shunt series were obtained at this time, with normal radiologic findings. Initial laboratory results are summarized in Table 1 and demonstrate a neutrophilic leukocytosis, a pre-renal pattern of acute kidney injury, and urinalysis showing bacteriuria with pyuria, consistent with acute UTI. He was admitted to an inpatient service, where empiric ceftriaxone (2 g/day) was continued pending culture and susceptibility results, consistent with standard initial management for pediatric febrile UTIs.

**Table 1:** Laboratory results obtained in the Emergency Department at the time of admission Abnormal quantitative results are labeled as High/Low (H/L), and qualitative results as Positive/Negative, with abnormal values shown in bold. UA, urinalysis; hpf, high-power field

Laboratory Parameter	Result	Normal Range
White Blood Cells	11.4 H	3.8-10.4 x 10^9^/L
Red Blood Cells	4.37	4.20-5.30 x 10^12^/L
Hemoglobin	12.6	12.4-15.7 g/dL
Hematocrit	36.4 L	38.0-47.0%
Mean Corpuscular Volume	83.3	79.9-93.0 fL
Mean Corpuscular Hemoglobin	28.8	25-35 pg
Red Cell Distribution Width Std dev	42.2	35-52 fL
Platelets	324	117-381 x 10^9^/L
Neutrophils	10.58 H	1.40-6.10 x 10^9^/L
Lymphocytes	0.59 L	1.00-3.20 x 10^9^/L
Monocytes	0.06 L	0.20-0.80 x 10^9^/L
Eosinophils	0.01 L	0.10-0.20 x 10^9^/L
Basophils	0.02	0-0.10 x 10^9^/L
Sodium (Na^+^)	139	136-146 mEq/L
Potassium (K^+^)	4.1	3.5-5.1 mEq/L
Chloride (Cl^-^)	104	98-110 mEq/L
Bicarbonate (HCO_3_^-^)	20 L	22-28 mEq/L
Urea Nitrogen	26 H	7-18 mg/dL
Creatinine	1.10	0.6-1.2 mg/dL
Glucose (random, non-fasting)	108	<140 mg/dL
Lactic Acid	1	<2 mEq/L
Bilirubin (total)	0.6	0.1-1.0 mg/dL
Alkaline Phosphatase	139	44-147 U/L
Alanine Aminotransferase	11	10-40 U/L
Aspartate Aminotransferase	18	12-38 U/L
C-Reactive Protein	112 H	0.0-4.9 mg/L
Procalcitonin	0.54 H	<0.1 ng/mL
UA Color	Yellow	Yellow
UA Appearance	Turbid	Clear
UA pH	7	5.5-7.5
UA Specific Gravity	1.009	1.005-1.030
UA Glucose	62	<130 mg/dL
UA Ketones	Negative	Positive if >2.9 mg/dL
UA Blood	>25 H	<3 cells/µL
UA Protein	30 H	<5 mg/dL
UA Bilirubin	Negative	Positive if >0.5 mg/dL
UA Urobilinogen	0.8	0.1-1.0 mg/dL
UA Nitrite	Positive	Positive if >0.05 mg/dL
UA Leukocyte Esterase	>500 H	<5 cells/µL
UA White Blood Cells	>100 H	<5 cells/hpf
UA Red Blood Cells	11-20 H	<2 cells/hpf
UA Bacteria	>10000 H	<2 cells/hpf
UA Squamous Cells	1	<3 cells/hpf

Approximately 30 hours after admission, blood cultures flagged positive, and species identification confirmed *M. morganii*. A urine culture obtained prior to empiric ceftriaxone administration also grew *M. morganii*. The patient remained on ceftriaxone for an additional 24 hours while awaiting susceptibility results, as presented in Table 2. Repeat physical examination revealed no obvious source of infection, and follow-up blood cultures were obtained. Vital signs at this time were: T 38.1°C, HR 103 bpm, BP 109/64 mmHg, RR 20, and SpO₂ 98% on room air. Although the organism was reported as sensitive to ceftriaxone, the patient was switched to IV cefepime (2 g twice daily for 14 days) at the recommendation of in-house infectious disease specialists, due to *M. morganii*’s known resistance patterns and the potential for AmpC-mediated beta-lactamase expression.

**Table 2:** Antibiotic susceptibility testing for Morganella morganii isolate

Antibiotic	Minimum Inhibitory Concentration (µg/mL)	Susceptibility Interpretation
Amoxicillin/Clavulanate	>16/8	Resistant
Ampicillin	>16	Resistant
Ampicillin/Sulbactam	>16/8	Resistant
Cefazolin	>16	Resistant
Cefepime	<=2	Susceptible
Ceftazidime	<=1	Susceptible
Ceftriaxone	<=1	Susceptible
Ciprofloxacin	1	Resistant
Ertapenem	<=0.5	Susceptible
Imipenem	4	Resistant
Levofloxacin	1	Intermediate
Meropenem	<=1	Susceptible
Piperacillin/Tazobactam	<=16	Susceptible
Trimethoprim/Sulfamethoxazole	>2/38	Resistant

**Figure 1:**
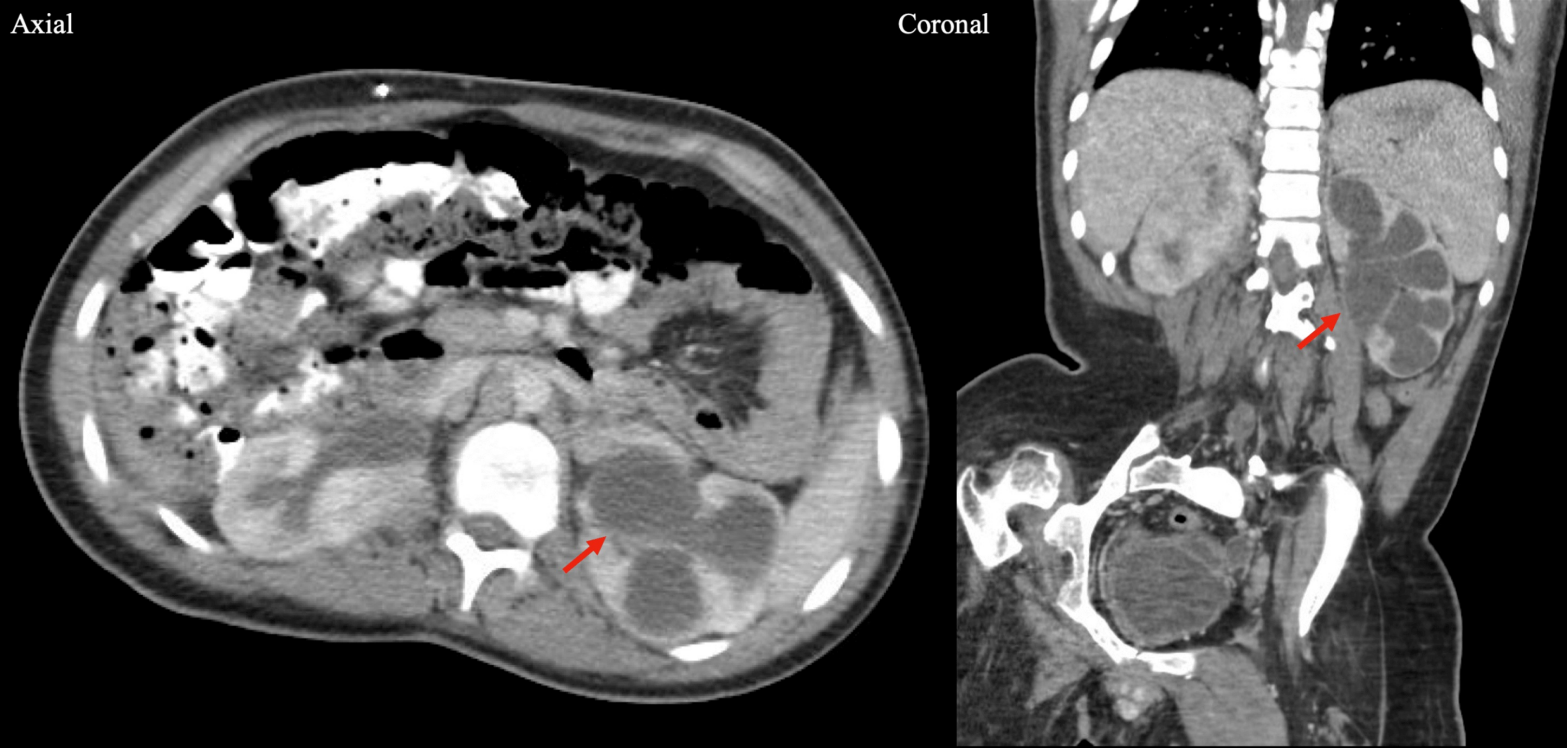
Axial and coronal CT scan of the abdomen and pelvis Axial and coronal CT scan of the abdomen and pelvis, demonstrating left-sided hydronephrosis characterized by dilation of the renal pelvis and calyces (arrows), with associated diminished parenchymal attenuation concerning for early pyelonephritis. CT, computed tomography

Rising blood urea nitrogen (BUN) and creatinine prompted evaluation for possible acute kidney injury, and computed tomography (CT) of the abdomen and pelvis revealed severe left-sided hydronephrosis with early signs of pyelonephritis (Figure 1). Interventions included increased oral intake, maintenance fluids, and adjustment of bladder catheterization from every six hours to every three hours. BUN and creatinine peaked at 51 mg/dL and 1.49 mg/dL, respectively, three days after admission, and decreased to 31 mg/dL and 1.17 mg/dL over 72 hours, remaining stable thereafter. Baseline creatinine was 0.48 mg/dL one year prior, and 0.90 mg/dL four months prior. The remainder of the hospital course was uncomplicated. The patient was discharged home 10 days after admission to complete the remaining portion of his 14-day cefepime course via a peripherally inserted central catheter (PICC) line. Follow-up blood cultures at discharge were sterile, and a urology follow-up appointment was scheduled for the following week.

The patient was seen for outpatient follow-up one week after discharge and was clinically well, reporting complete resolution of his initial symptoms. Repeat urinalysis at this time was normal, with no pyuria, hematuria, or bacteriuria. Further blood cultures were not obtained, as the repeat cultures drawn during hospitalization remained sterile. Laboratory testing demonstrated a normal leukocyte count and stable renal function, with creatinine of 1.02 mg/dL. The patient completed the full 14-day course of cefepime via PICC without complications. Overall, he returned to his baseline functional status, with no residual sequelae of bacteremia or renal impairment.

## Discussion

In neonates, *M. morganii* bacteremia is typically observed in early-onset disease and may be related to maternal or perinatal factors [7-11]. In older children, the development of UTI and subsequent bacteremia is primarily influenced by structural and functional urinary tract factors, including bladder emptying and length of the urethra. Bacteremia in elderly adults is often reported in the context of geriatric immunosenescence [2,4,12]. In this case, bacteremia occurred in an immunocompetent 13-year-old, a rare presentation [12]; however, several recognized risk factors were present [2,4]. Specifically, the patient’s history of recurrent UTIs with drug-resistant pathogens, frequent infections, and underlying structural and functional urinary tract abnormalities placed him at high risk for bacteremia. Positive urinalysis findings and imaging demonstrating hydronephrosis with early pyelonephritis further support the urinary tract as the likely entry point for *M. morganii* into the bloodstream. This is consistent with reported adult risk factors, including renal disease [4]. Although literature reports mortality rates exceeding 20% [4], this data primarily reflects adult populations in whom age and comorbidities contribute significantly to outcomes. The favorable outcome in this patient may be attributed to his younger age, early recognition, and antibiotic management. In addition to biological risk factors, practical challenges in home-based care may have contributed to the frequency of UTIs in this patient. As the sole caregiver, the patient’s mother relied on handwashing and clean, reusable materials for intermittent catheterization, which aligns with standard practice in home settings but still carries inherent risk for infection. Supplies such as sterile gloves, lubricant, or single-use catheters were not consistently available, increasing vulnerability to infection. 

The rarity of *M. morganii* bacteremia in this demographic makes this case notable. The most current and comprehensive retrospective study, published in 2022, estimates the incidence of *Morganella* bacteremia at only 9.2 per million per year [4]. An older study, published in 2007, puts this estimate even lower, at only 3 per million per year [6]. Between both studies, a total of zero cases were reported for patients 1-19 years of age over a combined 25-year surveillance period. To our knowledge, only a single paper, published in 2019, reports on *M. morganii* bacteremia in this age group, with six cases documented between 1997 and 2014 [12]. This makes the current report among the few documented instances in the literature.

Given *M. morganii*’s role as a uropathogen, particularly in patients with neurogenic bladder, chronic catheter use, or recurrent/structural urinary tract anomalies, clinicians should remain alert to the possibility of this infection in similar pediatric populations. When evaluating children with fever and urinary symptoms, differentials such as *E. coli*, *Klebsiella*, *Proteus*, and *Enterococcus* should also be considered, as they still remain more common causes of bacteremia in this setting. Although the urinary tract was the most likely source in this patient, *M. morganii* is also part of the normal intestinal flora. In children with ventriculoperitoneal shunts, gut translocation has been reported as a pathway to shunt infection, peritonitis, and secondary bacteremia [12]. While not likely in this patient, these alternative routes are important considerations for clinicians managing children with different anatomic or device-related risk factors.

Even though the patient’s infection was managed successfully, awareness of this pathogen is key because of its resistance profile. *M. morganii* exhibits intrinsic resistance via its inducible AmpC β-lactamase, conferring resistance to ampicillin, amoxicillin, and many early-generation cephalosporins [13]. While surveillance studies have not demonstrated a clear increase in novel resistance patterns [4], broader literature reflects a concerning trend that clinical isolates increasingly harbor mobile resistance genes, including carbapenemases (e.g., blaNDM-1, blaNDM-5, blaOXA-181), ESBLs, and integrons [13-15]. This expanding mobilome underscores the importance of clinical vigilance, particularly in vulnerable patients. Previous literature has also noted limitations due to sample size and the relatively sparse reporting across multiple centers in the already limited body of work detailing the epidemiology of serious *M. morganii* infections [2-4,12]. This case report adds to the limited literature on pediatric *Morganella* bacteremia and may contribute to a better understanding of its incidence, epidemiology, and clinical management.

## Conclusions

While the clinical course of this patient was uncomplicated, the occurrence of *M. morganii* bacteremia in an immunocompetent 13-year-old emphasizes the importance of clinician awareness of this uncommon pathogen. Although rare in children, this organism carries a rising potential for antimicrobial resistance, and, with very few documented pediatric instances, this report contributes to the limited literature on *M. morganii* bacteremia and may help inform clinical recognition and management.
